# Efficacy of therapeutic suggestions under general anesthesia: a systematic review and meta-analysis of randomized controlled trials

**DOI:** 10.1186/s12871-016-0292-0

**Published:** 2016-12-22

**Authors:** Jenny Rosendahl, Susan Koranyi, Davina Jacob, Nina Zech, Ernil Hansen

**Affiliations:** 1 Institute of Psychosocial Medicine and Psychotherapy, Jena University Hospital, Friedrich-Schiller University, Stoystr. 3, 07743 Jena, Germany; 2 Department of Medical Psychology and Medical Sociology, University Hospital Leipzig, Philipp-Rosenthal-Str. 55, 04103 Leipzig, Germany; 3 Department of Anaesthesiology, University Hospital Regensburg, Franz-Josef-Strauss-Allee 11, 93053 Regensburg, Germany

**Keywords:** Therapeutic suggestions, Anesthesia, general, Efficacy, treatment, Meta-analysis

## Abstract

**Background:**

General anesthesia does not block central nervous processing of auditive information. Therefore, positive suggestions even given during surgery might have the potential to encourage well-being and recovery of patients.

Aim of this review was to summarize the evidence on the efficacy of therapeutic suggestions under general anesthesia in adults undergoing surgery compared to an attention control (i.e. white noise).

**Methods:**

We included randomized controlled trials that investigated therapeutic suggestions presented during general anesthesia to adult patients undergoing surgery or medical procedures. Outcomes on pain intensity, mental distress, recovery, use of medication, measured postoperatively within hospitalization were considered. Electronic searches were carried out in the following databases (last search February 23, 2015): MEDLINE, CENTRAL, Web of Science, PsycINFO, ProQuest Dissertations and Theses.

**Results:**

Thirty-two eligible randomized controlled trials were included, comprising a total of 2102 patients. All studies used taped suggestions. Random effects meta-analyses revealed no effects on pain intensity (Hedges’ g = 0.04, CI 95% [−0.04; 0.12], number needed to treat [NNT] = 44.3) and mental distress (g = 0.03, CI 95% [−0.11; 0.16], NNT = 68.2). In contrast, we found small but significant positive effects on use of medication (g = 0.19, CI 95% [0.09; 0.29], NNT = 9.2) and on recovery (g = 0.14, CI 95% [0.03; 0.25], NNT = 13.0). All effects were homogeneous and robust.

**Conclusions:**

Even though effects were small, our results provide indications that intraoperative suggestions can have the potential to reduce the need for medication and enhance recovery. Further high quality trials are needed to strengthen the promising evidence on the efficacy of therapeutic suggestions under general anesthesia for patients undergoing surgery.

**Electronic supplementary material:**

The online version of this article (doi:10.1186/s12871-016-0292-0) contains supplementary material, which is available to authorized users.

## Background

Recovery from anesthesia and surgery is often hampered by side effects such as pain or postoperative nausea and vomiting (PONV), by disturbances of well-being and even complications. Pain and PONV are usually prevented and treated by medications that carry their own side effects. Other challenges such as anxiety, hopelessness and negative expectations further impair recovery and outcome [1], or lead to nocebo effects [2] which usually cannot be treated with drugs and call for non-pharmacological approaches.

Among psychological interventions to improve recovery and well-being hypnotherapeutic approaches are most effective [3]. Several meta-analyses show small to large effect sizes of therapeutic suggestions given pre- or postoperatively with or without hypnosis induction on various outcomes [3–6].

Some of the studies included suggestions presented during general anesthesia to the unconscious patient [5, 6]. In this context, suggestions are defined “as verbal or nonverbal messages that the receiver involuntarily accepts and follows” [7] and that might affect emotions, behavior and autonomous body functions. This approach is based on the consideration that anesthesia does not interrupt perception of sounds and words by the brain [8]. Intraoperative measurement of auditory evoked potentials has shown that the central auditory pathway remains intact during general anesthesia [9, 10]. Even further processing of words in the central nervous system including development of memory and appropriate responses has been demonstrated by postoperative recognition of intraoperatively presented words [11, 12], and postoperative nonverbal responses to instructions given during anesthesia [13–15]. In some cases, intraoperative awareness occurs under general anesthesia with explicit memory of the situation and of conversations [16]. In addition, the occurrence of implicit memory has been proven much more frequently [17]. Moreover, strong impact of negative intraoperative remarks on prognosis has been reported [18, 19].

One meta-analysis so far investigated the efficacy of therapeutic suggestions presented during general anesthesia to encourage well-being and recovery of surgical patients and has found mixed results [20]. Even though the effect on postoperative hospitalization was not statistically significant, the small positive effect of suggestions on patient-controlled analgesia reached statistical significance. However, these results must be interpreted with caution since a) the inclusion of non-randomized trials threatens the validity of meta-analytic results and b) the effects on patient-controlled analgesia are based on four studies only.

Hence, the present meta-analysis investigates the efficacy of therapeutic suggestions under general anesthesia on surgically relevant postoperative outcomes, i.e., pain intensity, mental distress, recovery, or the use of medication, and intraoperative outcomes, i.e., length of procedure and physiological parameters, by including randomized controlled trials only.

## Methods

Objectives, inclusion criteria, and methods have been pre-specified in a review protocol [21].

### Identification and selection of studies

Eligible studies were randomized controlled trials that investigated therapeutic suggestions presented during general anesthesia to adult patients undergoing surgery or medical procedures. If the intervention group received a combination of therapeutic suggestions and another psychological intervention or if therapeutic suggestions were not solely implemented intraoperatively, the study was excluded. Eligible control groups were “treatment as usual” (defined as the standard surgical care policy of the hospital) and “attention control” groups (defined as providing same amount of time and attention in addition to standard surgical care; e.g., blank tape, white noise). The included trials reported on at least one of the following outcomes measured via self- and/or observer reports: pain intensity, mental distress, recovery, use of medication, measured postoperatively within hospitalization. In addition, intraoperative outcomes, i.e., length of procedure and physiological parameters, were included (Additional file 1: Table S1).

Deviating from the protocol [21], we did not limit study inclusion to trials with a sample size of at least 20 participants in each trial arm, but rather tested this restriction in sensitivity analyses.

Electronic searches were carried out in the following databases (last search February 23, 2015): MEDLINE, CENTRAL, Web of Science, PsycINFO according to a search strategy that specified terms referring to the patient population (e.g., surg$.ti.ab.kw, General Surgery/, Anesthesia. General/), treatment (e.g., suggestion$.ti.ab.kw, Suggestion/), and study design (e.g., randomized controlled trial.pt). The search strategy was developed with consideration of validated search strategies for retrieving randomized controlled trials [22]. The MEDLINE search strategy is shown in Appendix. We adapted the strategy for the Cochrane Central Register of Controlled Trials (CENTRAL), Web of Science and PsycINFO.

In order to identify further trials, lists of references of relevant articles and previous reviews were also checked. Additionally, we screened ProQuest Dissertations and Theses Full Text Database to identify any unpublished material. One author (DJ) screened titles and abstracts of database records and retrieved full texts for eligibility assessment.

### Data extraction and management

A pilot-tested data extraction form was used to collect the following information from eligible trials: characteristics of patients, intervention, control group, outcomes, bibliographic information, and effect size related data.

Data were independently extracted by two raters (DJ, JR). Inter-rater disagreement was resolved through consensus. In case of missing information, study authors were contacted. If information on effect sizes was missing and could not be retrieved, data had to be approximated using different estimation methods (e.g., estimating statistics from graphs without numerical data, setting an effect size to zero if non-significant results were mentioned without reporting statistical parameters).

### Assessing the risk of bias in included studies

To assess risk of bias in the included studies, common markers of internal validity from the Cochrane Risk of Bias Tool were extracted [23]. The risk of bias assessment was conducted by two independent raters (DJ, SK) who were previously trained and blinded to extracted effect size estimates. Disagreements were resolved by discussion with one author (JR). Inter-rater agreement for the risk of bias assessment using Cohen’s kappa (*κ*) was excellent, *κ* = 0.76 [24].

### Summary measures

Corrected standardized mean differences (Hedges’ g) were calculated for each assessment time-point and measurement multiplied by a small sample bias correction factor [25]. An effect size of 0.5 thus indicates that the mean of the experimental group is half a standard deviation larger than the mean of the control group. The magnitude of Hedges’ g was interpreted within the same ranges as Cohen’s d, regarding 0.20, 0.50, and 0.80 as small, medium, and large effect sizes, respectively [26]. Since such effect sizes are generally not easy to interpret in terms of clinical significance, effect sizes Hedges’ g were transformed into numbers needed to treat (NNT) [27]. For all dichotomous outcomes, Log Odds Ratios were computed and converted to Hedges’ g [28] in order to pool across different effect size formats.

If a study comprised more than one intervention group [29–31] the shared control group was divided out approximately evenly among the comparisons [32].

### Data synthesis

Outcome data were meta-analyzed using a random-effects approach. The generic inverse variance method was applied with heterogeneity estimated using the DerSimonian-Laird method [33]. Statistical heterogeneity between trials was assessed with χ^2^ heterogeneity tests (Cochran’s Q) and I^2^ statistic [34]. I^2^ describes the percentage of the variability in effect estimates that is due to heterogeneity rather than chance, with values from 0 to 40% indicating no important heterogeneity, 30 to 60% moderate, 50 to 90% substantial, and 75 to 100% considerable heterogeneity, respectively [35].

### Risk of bias across studies

In order to test for publication bias funnel plots were inspected visually and the Egger test was run [36]. Additionally, Duval & Tweedie’s trim and fill procedure was used to obtain an adjusted estimate of the treatment effect after the publication bias had been taken into account and to indicate how many missing trials have been imputed to correct for publication bias [37].

### Additional analyses

We conducted sensitivity analyses in order to test the robustness of findings, examining if meta-analytic results change when excluding approximated effect sizes and when excluding small samples (*n* ≤ 20 per group). Moderator analyses were planned to explain statistical heterogeneity [38]. However, heterogeneity was not important (I^2^ < 40%). Therefore, we conducted stratified analyses in order to exploratory examine potential moderators.

All data analyses were performed using Comprehensive Meta-Analysis (CMA; Version 2.0; Biostat Inc.).

## Results

### Study selection

**Fig. 1:**
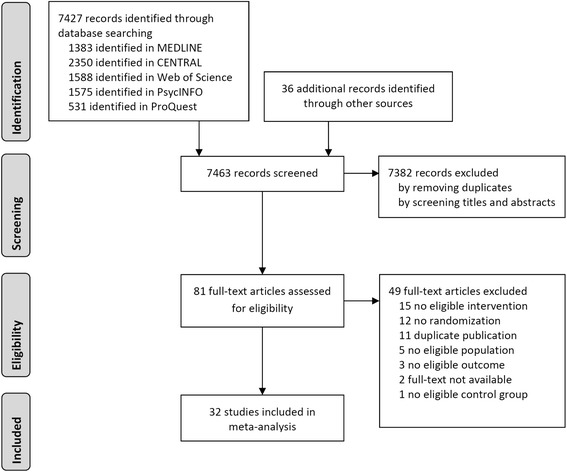
Flow chart of the study selection process

A total of 7427 records was screened and *N* = 32 randomized controlled trials were included in the meta-analysis. Figure 1 contains a flow chart of the study selection process.

### Description of included studies

Table 1 presents selected study characteristics. The majority of primary studies were published between 1986 and 2001; only one study [39] was published much earlier. Among the primary studies, there were three unpublished dissertations. One study was reported in German [40], all others were written in English. Altogether, *n* = 32 randomized controlled trials provided k = 37 comparisons between an intervention and a control group, incorporating a total of *n* = 1111 patients in intervention groups (M = 30.0, SD = 18.2) and *n* = 991 patients in control groups (M = 31.0, SD = 18.1). The mean age of patients in the intervention groups was 47.7 years (SD = 8.2), similarly in the control groups 47.2 years (SD = 9.4). The mean percentage of male patients was 17% (SD = 28.1) in intervention groups, and 17% (SD = 29.3) in control groups as well. This low percentage of male patients can be ascribed to a high proportion of studies including patients undergoing gynecological surgery; 16 primary studies investigated female patients only. In the majority of primary studies anesthesia was performed as “balanced anesthesia” with an opioid and an inhalational anesthetic (Table 1). In six studies, neuroleptanesthesia was used and in two studies total intravenous anesthesia (TIVA) with propofol or midazolam, respectively. Nitrous oxide was included in all except one study. In seven studies a benzodiazepine was applied for premedication. Therapeutic suggestions were presented via tape in all studies, played throughout the surgery in almost every study. Suggestion were judged as affirmative (e.g., “You will feel fine after the operation.”) in 12 intervention groups (32%), as non-affirmative (e.g., “After the operation you will *not* feel any nausea.”) in one (3%), and both affirmative and non-affirmative in 14 intervention groups (38%; no information reported for 10 intervention groups). In 19 intervention groups (51%), suggestion were accompanied by or alternated with soothing music or sounds. In all studies, the effects of therapeutic suggestions were compared against attention control. 18 studies (56%) used blank tapes/white noise, 7 studies (22%) offered sounds or music, and another 7 studies used spoken text (history of hospital, story of Peter Pan, parts of a cookery book) as control condition.

**Table 1:** Characteristics of the included studies

First author (publication year)	Country	Surgical procedure	Description of therapeutic suggestions ^a^	*n*	Description of control group	*n*	Anesthesia	Outcomes
Bethune et al. (1993) [47]	UK	Coronary artery bypass grafting	Positive; based on text used by Evans & Richardson (1988) [48]; music at the beginning	16	Blank tape with musical leader	17	I	Mental distress (mood, anxiety) Pain intensity Recovery (nausea, mobility, general recovery) Length of procedure
Block et al. (1991) [15]	USA	Operation on the fallopian tubes, vertical banding gastroplasty, total abdominal hysterectomy, ovarian cystectomy, cholecystectomy, myomectomy	Smooth, rapid recovery during a short post-operative stay; no pain, nausea or vomiting; rapid return of bowel and bladder function; rapid healing and mobility; comfort; relaxation; good appetite, sleep, mood, and feeling	109	Blank tape	100	I	Medication (antiemetics, Analgesics) Length of procedure
Boeke et al. (1988) [49]	NL	Elective cholecystectomy	Relaxation, wellbeing, pleasant feeling in every respect, absence of nausea and vomiting, good recovery; with seaside sounds	24	Nonsense suggestions with seaside sounds	26	N	Mental distress (well-being) Pain intensity Recovery (nausea, general recovery) Length of procedure Physiological parameters (blood loss)
Bonke et al. (1986) [50]	NL	Biliary tract surgery	Relaxation, well-being, comfortable feelings in every respect, lack of nausea or vomiting, no difficulty with bladder function or bowel movement, rapid recovery	31	Monotonous low-frequency noise, resembling the sound of a vacuum cleaner	30	N	Mental distress (well-being, relaxation) Pain intensity Medication (analgesics) Recovery (PONV, general recovery) Length of procedure Physiological parameters (blood loss)
Caseley-Rondi et al. (1994) [51]	Canada	Elective total abdominal hysterectomy and/or bilateral salpingo oophorectomy	Simple and positive; alternating with Japanese melodies	48	Blank tape alternating with Japanese melodies	48	I	Mental distress (mood, anxiety) Pain intensity Medication (analgesics) Recovery (nausea, general recovery)
Cowan et al. (2001) [52]	USA	Bariatric surgical patients	Positive; cognitive-behavioural approach: reinforcement of information received preoperatively, encouragement of performance of recovery regimes, positive interactions with personnel, improved self-image, relaxation	10	Blank tape	17	I	Recovery (turning, coughing, breathing, mobility)
Dawson et al. (2001) [29]	UK	Total abdominal hysterectomy with/without bilateral salpingo oophorectomy	(1) No pain (2) No sickness (3) No sickness, no pain	35 34 34	White noise	35	N (no N_2_O)	Pain intensity Medication (antiemetics, analgesics) Recovery (PONV) Length of procedure
De Houwer et al. (1996) [53]	Belgium	Coronary artery bypass grafting	Relaxation, fast recovery; based on Bonke et al. (1986) [50]; with background relaxing music	19	Blank tape	21	T	Mental distress (mood, anxiety) Recovery (general recovery) Length of procedure Physiological parameters (blood pressure, noradrenalin, adrenalin)
Eberhart et al. (1998) [54]	Germany	Thyroidectomy	Smooth postoperative recovery; relaxation, security, absence of nausea and vomiting	36	Blank tape	35	N	Medication (antiemetics, analgesics) Recovery (PONV) Length of procedure
Evans & Richardson (1988) [48]	UK	Total abdominal hysterectomy	Direct (no sickness, no pain), third person (operation is going very well and the patient is fine), description of normal postoperative procedures and coping advices	19	Blank tape	20	I	Mental distress (anxiety, mood) Pain intensity Recovery (PONV, pyrexia, bowel difficulties, flatulence, mobility, micturation, general recovery, complications) Length of procedure Physiological parameters (blood loss)
Furlong (1990) [55]	USA	Abdominal gynecological surgery	Positive	10	Blank tape	9	I	Mental distress (mood) Medication (analgesics) Length of procedure
Furlong & Read (1993) [56]	USA	Gynecological surgery or mastectomy	Positive; with background music	52	Blank tape	56	I	Mental distress (anxiety) Pain intensity Medication (analgesics) Recovery (PONV, mobility, bowel difficulties, micturition, wound healing)
Jelicic et al. (1993) [30]	NL	Cholecystectomy	(1) Affirmative (relaxation, comfort, quick healing) and non-affirmative (absence of tension, nausea); alternating with seaside sounds (2) Affirmative (3) Non-affirmative	21 20 20	Irrelevant text, i.e. excerpts from a cookery book	21	N	Mental distress (well-being) Length of procedure Physiological parameters (blood loss)
Korunka et al. (1992) [40]	Austria	Hysterectomy	Positive; followed by indirect (general wellbeing, absence of pain)	55	Operation sounds	53	I	Pain intensity Medication (analgesics) Length of procedure
Lebovits et al. (1999) [57]	USA	Elective hernia repair	Positive postoperative course, well-being and relaxation, minimal side effects, no difficult voiding, minimal discomfort such as sore throat, muscle aches and emetic symptoms; requirement of minimal pain medication	34	History of hospital	36	T	Pain intensity Medication (analgesics) Recovery (PONV, side effects) Length of procedure
Liu et al. (1992) [58]	UK	Total abdominal hysterectomy	Affirmative and non-affirmative (comfort and rapid recovery; absence of pain or feelings of sickness)	24	History of hospital	25	I	Mental distress (mood) Pain intensity Medication (analgesics) Recovery (nausea, pyrexia, wound healing, mobility, flatulence) Length of procedure Physiological parameters (blood loss)
Liu et al. (1993) [59]	UK	Surgical repair of fractured neck of femur	Positive (relating to comfort, mobility, nausea, general recovery and discharge after surgery)	58	History of hospital	61	GA	Pain intensity Medication (analgesics, antiemetics) Recovery (ADL, mobility, pyrexia)
Maroof et al. (1997) [60]	Saudi Arabia	Elective abdominal hysterectomy	Positive (good progress, well-being after surgery, absence of sickness)	25	Blank tape	25	I	Medication (antiemetics) Recovery (PONV, fluid replacement) Length of procedure
Mastropietro (1998) [31]	USA	Open gynaecological procedures through a midline incision	(1) Synchronized with soothing music presented when stress test was positive (comfortable, relaxed intraoperative and postoperative experience) (2) Randomly presented with soothing music (comfortable, relaxed intraoperative and postoperative experience)	24				
McLintock (1990) [61]	UK	Elective abdominal hysterectomy by Pfannenstiel’s incision	Positive (positive progress, feelings of warmth, comfort, calmness and relaxation; absence of pain)	30	Blank tape	30	I	Pain intensity Medication (analgesics) Recovery (PONV)
McWilliams (1990) [62]	USA	Lumbar laminectomy surgery	Suggestions interspersed with environmental sounds	30	Environmental sounds alone	30	GA	Medication (analgesics, antiemetics) Recovery (general recovery)
Melzack et al. (1996) [63]	Canada	Cholecystectomy or hyterectomy	Positive; regarding the recovery process (e.g., feeling fine, mild pain only); interspersed with music	10	Excerpts from physiology book	10	I	Pain intensity Length of procedure
Moix et al. (1996) [64]	Spain	Abdominal hysterectomy	Affirmative (feeling of relaxation during surgery, good progress, easy and rapid recovery, rapid mobility, comfort, good appetite, digestion, micturition, sleep and mood)	14	Monotonous sounds	13	I	Pain intensity Medication (analgesics) Recovery (vomiting, pyrexia, digestion, appetite, sleep, bowel function, general recovery, micturition, complications) Length of procedure Physiological parameters (blood pressure, heart rate)
Münch & Zug (1990) [65]	Germany	Thyroidectomy	General (comfort and well-being), direct (relaxing and feeling well, no vomiting, no nausea, only little pain); classical music played in a modern fashion	18	Blank tape	18	N	Mental distress (well-being) Pain intensity Recovery (PONV) Length of procedure
Nilsson et al. (2001) [66]	Sweden	Hysterectomy	Relaxing and encouraging; music (relaxing and calming; accompanied by soothing sounds of sea waves)	31	Operation room sounds	28	I	Mental distress (well-being) Pain intensity Medication (analgesics) Recovery (nausea, fatigue, mobility) Length of procedure Physiological parameters (blood loss)
Oddby-Muhrbeck et al. (1995) [67]	Sweden	Elective breast surgery	Affirmative (safe atmosphere, quick recovery, feelings of hunger and thirst)	35	Low electronic sound set	35	I	Pain intensity Medication (analgesics) Recovery (PONV, general recovery) Length of procedure Physiological parameters (blood loss)
Pearson (1961) [39]	USA	Thyroidectomy, gastrectomy, hernia repair, hysterectomy, pelvic laparoscopy, vein stripping	In a permissive manner (relaxation and selfresponsibility of the patient for the course of recovery)	43	Blank tape	38	GA	Medication (analgesics) Recovery (general recovery)
Rosenberg (1992) [68]	USA	Hysterectomy, myomectomy, other gynaecological procedures, cholecystectomy	Positive (smooth, comfortable, and rapid recovery, absence of sickness and pain), procedural/ sensory information about course of surgery and recovery; imagery, recommendations for dealing with postoperative events and sensations	33	Blank tape	32	I	Mental distress (anxiety) Pain intensity Medication (analgesics, antiemetics) Recovery (mobility, pyrexia, micturation) Length of procedure
Steinberg et al. (1993) [69]	USA	Total abdominal hysterectomy or breast reconstruction by transverse rectus abdominus musculocutaneous reconstruction flap	Positive (relating to nausea and anxiety)	30	Blank tape	30	I	Mental distress (anxiety) Pain intensity Medication (analgesics) Recovery (PONV)
van der Laan et al. (1996) [70]	USA	Hysterectomy, myomectomy, gynecologic laparotomy	Nonspecific affirmative (feelings of relaxation and security)	20	Story of Peter Pan	20	I	Mental distress (anxiety) Pain intensity Medication (analgesics) Recovery (nausea) Length of procedure
Williams et al. (1994) [42]	UK	Major gynaecological surgery	Positive (smooth and uncomplicated progress of surgery, well-being after surgery, absence of sickness)	22	Blank tape	29	I	Medication (analgesics) Recovery (PONV, fluid replacement) Length of procedure Physiological parameters (blood loss)
Woo et al. (1987) [71]	USA	Hysterectomy	Positive (rapid recovery)	7	Ocean sounds	7	I	Medication (analgesics)

*NL* Netherlands, *I* inhalation anesthesia, *N* neuroleptanesthesia, *T* intraveneous anesthesia, *GA* general anesthesia (not specified), *PONV* postoperative nausea and vomiting; a as reported in primary study

Additional file 2: Table S2 contains information on the risk of bias in included studies. Overall, the risk of bias in the included studies was mainly judged as low; no study indicated a high risk of bias in any quality item. However, due to missing information in the studies a high percentage of items was judged as unclear.

### Meta-analytic results

Across all included postoperative outcomes, there was a small, but statistically significant and homogeneous effect of therapeutic suggestions compared to attention control (g = 0.13, 95% CI [0.04; 0.23], k = 37, *p* = .005; I^2^ = 0%).

**Fig. 2:**
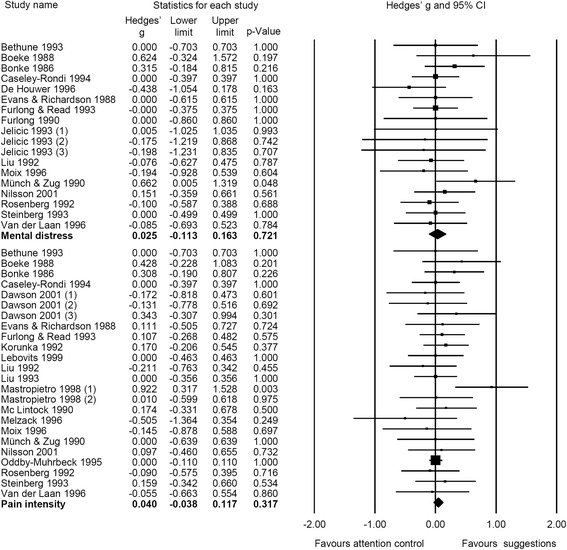
Forest plot of meta-analytic results for mental distress and pain intensity

**Fig. 3:**
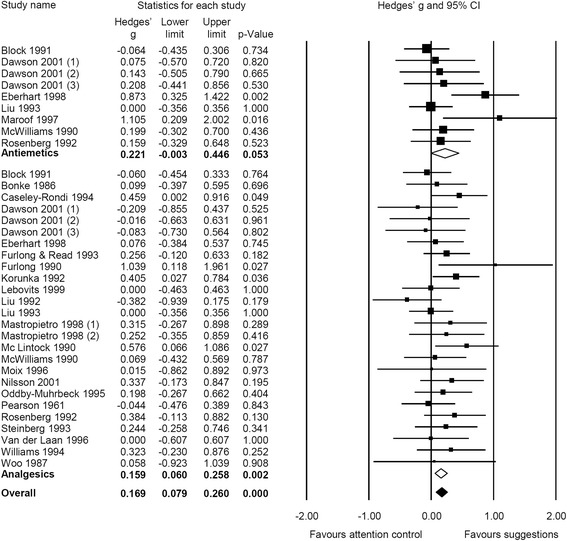
Forest plot of meta-analytic results for medication, stratified for use of antiemetics and analgesics

**Fig. 4:**
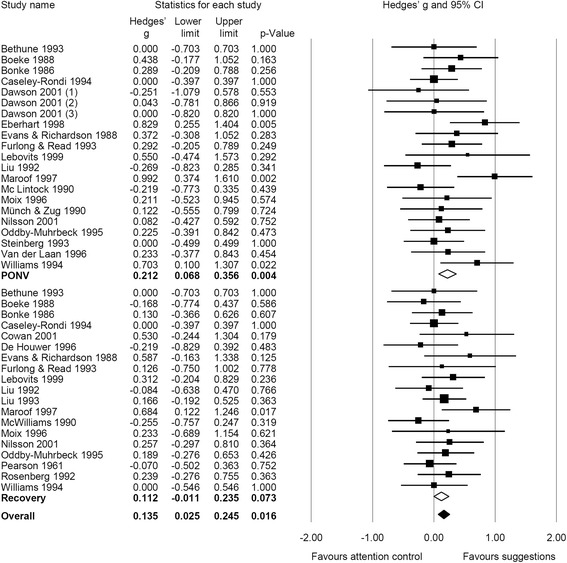
Forest plot of meta-analytic results for recovery, stratified for postoperative nausea and vomiting (PONV) and recovery (all other outcomes)

When outcomes were analyzed separately, we found effects of therapeutic suggestions on pain intensity (g = 0.04, CI 95% [−0.04; 0.12], NNT = 44.3) and mental distress (g = 0.03, CI 95% [−0.11; 0.16], NNT = 68.2) to be close to zero and non-significant. However, small significant effects in favor of therapeutic suggestions appeared on medication use (g = 0.19, CI 95% [0.09; 0.29], NNT = 9.2) and on recovery (g = 0.14, CI 95% [0.03; 0.25], NNT = 13.0). Stratifying analyses on medication use and recovery with respect to outcomes, we found small, significant effects for therapeutic suggestions on PONV (g = 0.21, CI 95% [0.07; 0.36], NNT = 8.3) and analgesic use (g = 0.16, CI 95% [0.06; 0.26], NNT = 11.0). Therapeutic suggestions also revealed a small effect on antiemetic use (g = 0.22, CI 95% [−0.003; 0.45], NNT = 7.9) and on all other recovery outcomes (g = 0.11, CI 95% [−0.01; 0.24], NNT = 15.6), even though these effects were marginally significant only (Figs. 2, 3, and 4). Heterogeneity for all outcomes was not important (I^2^ < 40%).

Regarding intraoperative outcomes, therapeutic suggestions revealed a small effect on physiological parameters, even though this effect was not significant (g = 0.13, CI 95% [−0.16; 0.42], k = 12, *p* = .389; I^2^ = 62%). Effects of therapeutic suggestions on length of surgical procedure (g = −0.04, CI 95% [−0.14; 0.07], k = 28, *p* = .499; I^2^ = 0%) were close to zero and non-significant.

### Publication bias

A visual inspection of the funnel plot (see Additional file 3: Figure S1) gave no indication of publication bias as trials are distributed symmetrically around the pooled effect size. Egger’s test of funnel plot asymmetry did not indicate publication bias (t(35) = 0.18; *p* = .428), and Duval & Tweedie’s trim and fill procedure resulted in no trimmed studies. Hence, publication bias does not pose a threat to the accuracy of our meta-analytic results.

### Additional analyses

We tested the robustness of effects for primary outcomes. After excluding approximated effect sizes for all outcome categories the meta-analytic result patterns (size of effect estimates and significance) did not change considerably though effect sizes were slightly larger and reached significance for recovery. Furthermore, effects were robust against the exclusion of small samples (*n* ≤ 20 per group) yielding effect sizes comparable in size and (non-)significance (Additional file 4: Table S3).

Since heterogeneity was not important at all (I^2^ < 40%), we did not run our pre-specified subgroup analyses. However, in order to get some ideas about potential moderators we exploratory conducted stratified analyses for PONV and antiemetic use since for all other postoperative outcomes results were homogeneous (I^2^ = 0%). Studies applying suggestions related to the absence of PONV (e.g., “no sickness”) yielded larger effects than studies without such suggestions, but this difference was not significant for both outcomes. There was no indication of an association between treatment effects and affirmativity of suggestions. Furthermore, studies using neuroleptanesthesia did not differ from those with intravenous or inhalation anesthesia.

Stratifying the analyses according to risk of bias, we only found differences with respect to handling of incomplete outcome data which were significant by trend for PONV (*p* = .061) with studies evaluated as low risk bias yielding smaller effects than studies judged as unclear risk of bias. Random sequence generation had no influence of treatment effects (Additional file 5: Table S4).

## Discussion

The present meta-analysis aimed at evaluating the efficacy of therapeutic suggestions presented during general anesthesia to patients undergoing surgery or medical procedures. Currently, the efficacy of therapeutic suggestions applied under general anesthesia has been investigated on hospitalization and patient-controlled analgesia exclusively. Our meta-analysis expands this knowledge by adding results on pain intensity, mental distress, use of medication, and recovery.

We found small, significant positive effects of therapeutic suggestions on recovery and medication use which proved to be robust and free of publication bias. When analyzing outcomes in more detail, highest effects were found for PONV and analgesic use. Comparable results of therapeutic suggestions on the amount of morphine administered via patient-controlled analgesia were also reported in the meta-analysis of Merikle and Daneman [20]. However, there was no effect of therapeutic suggestions on pain intensity or mental distress.

One reason for the small or even zero effects might be the level of awareness. Usually, therapeutic suggestions were given during general anesthesia excluding the induction of anesthesia and emergence from anesthesia that are most sensitive to intraoperative awareness [16]. Another reason could be that when suggestions are presented via tape only, rapport and therapeutic relationship are missing, which are essential components of effective hypnosis or therapeutic suggestions [4, 8]. Accordingly, higher effect sizes of suggestions to reduce postoperative side effects spoken live compared to taped suggestions were reported [5, 6].

Since study effects were quite homogeneous, we merely ran stratified analyses on PONV and antiemetic use to get an idea about potential moderators of treatment effects. In this regard, the specificity of suggestions seems to have an influence on its efficacy since studies with specific PONV related suggestions yielded significant results on PONV, while studies with unspecific suggestions only resulted in non-significant effects. Thus, our results go along with studies demonstrating an impact of suggestion specificity on its efficacy [6].

Differences in anesthesia methods did not influence the efficacy of therapeutic suggestions, although neuroleptanesthesia is known to carry a higher risk of intraoperative awareness and lower interference with memory in comparison to balanced anesthesia with inhalational or intravenous anesthetics [16]. However, intraoperative awareness and memory are not considered a pre-requisite for effects of suggestions in unconscious patients [41, 8].

When interpreting these results the exploratory nature of the respective analyses should be considered. Although research on the impact of affirmativity and specificity of therapeutic suggestions on postoperative outcomes is available [6, 29, 30, 42] this issue has not been clarified conclusively. Studies examining the most efficacious phrasing of suggestions are still pending; an optimization of therapeutic suggestions is possible and needed.

Several limitations of the present meta-analysis are noteworthy. First, we excluded studies with children and studies where pre- or postoperative suggestions were presented in addition to those given intraoperatively. Both restrictions of inclusion might have led to smaller effects of suggestions during general anesthesia. There is some evidence of a higher level of efficacy of suggestive techniques in children [5], partly due to their higher suggestibility [43]. Moreover, meta-analytic findings have shown that suggestions are more effective when delivered at least in part prior to the medical procedure rather than solely during the medical procedure [5].

Second, the reporting quality, i.e. completeness and transparency, of the included studies was rather low making it difficult to adequately evaluate potential risks of bias. Particularly, methods of randomization and allocation concealment have been reported inadequately in the majority of studies, whereas blinding of participants, personnel, and outcome assessors was reported well. From the information on the anesthesia methods provided in the included studies no conclusion can be drawn on the precise depth of anesthesia and its impact on the results, besides that standard procedures were used without techniques to control depth, if reported, the dosage of anesthetics was reasonable, and the same procedure was used for intervention and control group. Finally, the latest available randomized controlled trial dates back to 2001.

It might be argued that insufficient anesthetic depth was more common at that time, but even modern electroencephalography (EEG)-based monitoring of anesthetic depth even could only reduce but not eliminate intraoperative awareness with recall (AWR) [44]. Current recommendations for AWR prevention include earplugs or music via earphones as an essential component. Positive suggestions should be considered as well, since being proposed for prophylaxis of posttraumatic stress disorder following AWR [45]. It has been claimed that effects of intraoperative suggestions are limited to insufficient depth of anesthesia [46], but even this pre-requisite is not absent in clinical practice today.

## Conclusions

Altogether, we found at least small overall effects of therapeutic suggestions, with no significant negative effect in any primary study. Hence, therapeutic suggestions could be a conceivable way to safely improve recovery and to reduce medication. In the light of the quite low effort and costs of implementation and use of suggestions it might be efficient to present suggestions under general anesthesia in clinical practice.

So far the evidence on the efficacy of therapeutic suggestions applied under general anesthesia has been summarized with respect to hospitalization and patient-controlled analgesia exclusively [20]. Our meta-analysis expands this knowledge by adding results on mental distress, pain intensity, medication, and recovery. With solely including randomized trials the internal validity of the findings should have been increased.

However, we cannot make clinical recommendations since the quality of evidence supporting the beneficial effects of therapeutic suggestions was rated as unclear in a considerable number of included trials, particularly with regard to selection bias and reporting bias. Moreover, there is a lack of respective publications after 2001. We encourage the proliferation of studies with a high methodological and reporting quality to strengthen the promising evidence for the efficacy of therapeutic suggestions presented during general anesthesia for patients undergoing surgery.

## Appendix

### Full electronic search strategy for PubMed (MEDLINE)

#1 suggestion$.ti.ab.kw

#2 Suggestion/

#3 hypno$.ti.ab.kw

#4 Hypnosis/

#5 Hypnosis. Anesthetic/

#6 Persuasive Communication/

#7 auditory information$.ti.ab.kw

#8 Intraoperative Care/ px [Psychology]

#9 1 OR 2 OR 3 OR 4 OR 5 OR 6 OR 7 OR 8

#10 exp Surgical Procedures. Operative/

#11 anesth$.ti.ab.kw

#12 anaesth$.ti.ab.kw

#13 Anesthesia/

#14 Anesthesia. General/

#15 Balanced Anesthesia/

#16 narcot$.ti.ab.kw

#17 narcosis.ti.ab.kw

#18 surg$.ti.ab.kw

#19 General Surgery/

#20 operat$.ti.ab.kw

#21 intraoperat$.ti.ab.kw

#22 intra-operat$.ti.ab.kw

#23 Intraoperative Period/

#24 intrasurg$.ti.ab.kw

#25 intra-surg$.ti.ab.kw

#26 medical procedure$.ti.ab.kw

#27 10 OR 11 OR 12 OR 13 OR 14 OR 15 OR 16 OR 17 OR 18 OR 19 OR 20 OR 21 OR 22 OR 23 OR 24 OR 25 OR 26

#28 randomized controlled trial.pt

#29 controlled clinical trial.pt

#30 randomized.ab

#31 placebo.ab

#32 randomly.ab

#33 trial.ti.

#34 28 OR 29 OR 30 OR 31 OR 32 OR 33

#38 9 AND 27 AND 34

## Additional files

Additional file 1: Table S1. Measures of outcomes. (DOCX 15 kb)
Additional file 2: Table S2. Risk of bias in individual studies. (DOCX 17 kb)
Additional file 3: Figure S1. Funnel plot of Hedges’ g against its standard error for all outcomes. (TIF 302 kb)
Additional file 4: Table S3. Results of sensitivity analyses for the exclusion of approximated effect sizes and effect sizes which were set to zero, and for the exclusion of small samples. (DOCX 18 kb)
Additional file 5: Table S4. Results of subgroup analyses for PONV and antiemetic use. (DOCX 20 kb)
Additional file 6: Data with descriptive and effect size related information. (XLSX 60.6 kb)

## Abbreviations

AWR: Awareness with recall
CENTRAL: Cochrane Central Register of Controlled Trials
EEG: Electroencephalography
NNT: Number needed to treat
PONV: Postoperative nausea and vomiting
TIVA: Total intravenous anesthesia

## References

[1] Eaker E, Pinsky J, Castelli WP (1992). Myocardial infarction and coronary death among women: psychosocial predictors from a 20- year follow-up of women in the Framingham Study. Am J Epidemiol.

[2] Häuser W, Hansen E, Enck P (2012). Nocebo phenoma in medicine: their relevance in everyday clinical practice. Dtsch Arztebl Int.

[3] Tefikow S, Barth J, Maichrowitz S, Beelmann A, Strauss B, Rosendahl J (2013). Efficacy of hypnosis in adults undergoing surgery or medical procedures: a meta-analysis of randomized controlled trials. Clin Psychol Rev.

[4] Montgomery GH, David D, Winkel G, Silverstein JH, Bovbjerg DH (2002). The effectiveness of adjunctive hypnosis with surgical patients: a meta-analysis. Anesth Analg.

[5] Schnur JB, Kafer I, Marcus C, Montgomery GH (2008). Hypnosis to manage distress related to medical procedures: a meta-analysis. Contemp Hypn.

[6] Kekecs Z, Nagy T, Varga K (2014). The effectiveness of suggestive techniques in reducing postoperative side effects: a meta-analysis of randomized controlled trials. Anesth Analg.

[7] Varga K (2013). Suggestive techniques connected to medical interventions. Interv Med Appl Sci.

[8] Ghoneim MM, Block RI (1992). Learning and consciousness during general anesthesia. Anesthesiology.

[9] Clark DL, Rosner BS (1973). Neurophysiologic effects of general anesthesia. Anesthesiology.

[10] Madler C, Keller I, Schwender D, Pöppel E (1991). Sensory information processing during general anaesthesia: effect of isoflurane on auditory evoked neuronal oscillations. Br J Anaesth.

[11] Millar K, Watkinson N (1983). Recognition of words presented during general anaesthesia. Ergonomics.

[12] Schwender D, Kaiser A, Klasing S, Peter K, Pöppel E (1994). Midlatency auditory evoked potentials and explicit and implicit memory in patients undergoing cardiac surgery. Anesthesiology.

[13] Bennett HL, Davis HS, Giannini AJ (1985). Non-verbal response to intraoperative conversation. Br J Anaesth.

[14] Goldmann L, Shah MV, Helden MW (1987). Memory of cardiac anaesthesia: psychological sequelae in cardiac patients of intraoperative suggestion and operating room conversation. Anaesthesia.

[15] Block RI, Ghoneim MM, Sum Ping ST, Ali MA (1991). Efficacy of therapeutic suggestions for improved postoperative recovery presented during general anesthesia. Anesthesiology.

[16] Mashour GA, Avidan MS (2015). Intraoperative awareness: controversies and non-controversies. Br J Anaesth.

[17] Sanders RD, Tononi G, Laureys S, Sleigh JW (2012). Unresponsiveness ≠ unconsciousness. Anesthesiology.

[18] Cheek DB (1964). Surgical memory and reaction to careless conversation. Am J Clin Hypn.

[19] Levinson BW (1965). States of awareness during general anaesthesia. Brit J Anaesth.

[20] Merikle PM, Daneman M (1996). Memory for unconsciously perceived events: evidence from anesthetized patients. Conscious Cogn.

[21] Jacob D, Tefikow S, Rosendahl J. Efficacy of therapeutic suggestions in adults undergoing surgery or medical procedures under general anaesthesia: a systematic review and meta-analysis of randomized controlled trials. PROSPERO 2013:CRD42013003963. http://www.crd.york.ac.uk/PROSPERO/display_record.asp?ID=CRD42013003963 [Assessed 18 Mar 2015].

[22] Lefebvre C, Manheimer E, & Glanville J. Searching for studies. In: Higgins JPT, Green S, eds. Cochrane handbook for systematic reviews of interventions version 5.1.0 (updated March 2011). The Cochrane Collaboration; 2011. Available from http://www.handbook.cochrane.org [Assessed 27 Apr 2015].

[23] Higgins JPT, Altman DG, Sterne JAC. Assessing risk of bias in included studies. In: Higgins JPT, Green S, eds. Cochrane handbook for systematic reviews of interventions version 5.1.0 (updated March 2011). The Cochrane Collaboration; 2011. Available from http://http://www.cochrane-handbook.org [Assessed 23 July 2014].

[24] Cicchetti DV (1994). Guidelines, criteria, and rules of thumb for evaluating normed and standardized assessment instruments in psychology. Psychol Assess.

[25] Hedges LV, Olkin I (1985). Statistical methods for meta-analysis.

[26] Cohen J (1992). A power primer. Psychol Bull.

[27] Kraemer HC, Kupfer DJ (2006). Size of treatment effects and their importance to clinical research and practice. Biol Psychiatry.

[28] Borenstein M, Hedges LV, Higgins JPT, Rothstein HR (2009). Introduction to meta-analysis.

[29] Dawson PR, Van Hamel C, Wilkinson D, Warwick P, O’Connor M (2001). Patient-controlled analgesia and intra-operative suggestion. Anaesthesia.

[30] Jelicic M, Bonke B, Millar K (1993). Effect of different therapeutic suggestions presented during anaesthesia on post-operative course. Eur J Anaesthesiol.

[31] Mastropietro CA (1998). Development and testing of a middle range theory of assessment and intervention for pain and awareness during anesthesia: efficacy of timing of a therapeutic suggestion for surgical pain relief [dissertation].

[32] Higgins JPT, Deeks JJ, Altman DG. Special topics in statistics. In: Higgins JPT, Green S, eds. Cochrane handbook for systematic reviews of interventions version 5.1.0 (updated March 2011). The Cochrane Collaboration; 2011. Available from http://www.handbook.cochrane.org [Assessed 27 Apr 2015].

[33] DerSimonian R, Laird N (1986). Meta-analysis in clinical trials. Control Clin Trials.

[34] Higgins JPT, Thompson SG, Deeks JJ, Altman DG (2003). Measuring inconsistency in meta-analyses. Br Med J.

[35] Deeks JJ, Higgins JPT, Altmann DG. Analysing data and undertaking meta-analyses. In: Higgins JPT, Green S, eds. Cochrane handbook for systematic reviews of interventions version 5.1.0 (updated March 2011). The Cochrane Collaboration; 2011. Available from http://www.handbook.cochrane.org [Assessed 24 Apr 2015].

[36] Egger M, Davey Smith G, Schneider M, Minder C (1997). Bias in meta-analysis detected by a simple, graphical test. Br Med J.

[37] Duval S, Tweedie R (2000). Trim and fill: a simple funnel-plot-based method of testing and adjusting for publication bias in meta-analysis. Biometrics.

[38] Thompson SG, Higgins JPT (2002). How should meta-regression analyses be undertaken and interpreted. Stat Med.

[39] Pearson RE (1961). Response to suggestions given under general anesthesia. Am J Clin Hypn.

[40] Korunka C, Guttmann G, Schleinitz D, Hilpert M, Haas R, Fitzal S (1992). Die Auswirkung von Suggestionen und Musik während Vollnarkose auf postoperative Befindlichkeit [The effects of suggestions and music presented during general anaesthesia on postoperative well-being]. Z Klin Psychol.

[41] Kihlstrom JF, Cork RC. Consciousness and anesthesia. In: Schneider S, Velmans M, editors. The Blackwell Companion to Consciousness. Chichester: Wiley; 2007. p. 628–39.

[42] Williams AR, Hind M, Sweeney BP, Fisher R (1994). The incidence and severity of postoperative nausea and vomiting in patients exposed to positive intra-operative suggestions. Anaesthesia.

[43] London P, Cooper LM (1969). Norms of hypnotic susceptibility in children. Develop Psychol.

[44] Palanca BJ, Mashour GA, Avidan MS (2009). Processed electroencephalogram in depth of anesthesia monitoring. Curr Opin Anaesthesiol.

[45] Bejenke CJ, Cyna A, Andrew MI, Tan SGM, Smith AF (2010). Intraoperative awareness. Handbook of communication in anaesthesia and critical care.

[46] Kerssens C, Ouchi T, Sebel PS (2005). No evidence of memory function during anesthesia with propofol or isoflurane with close control of hypnotic state. Anesthesiology.

[47] Bethune DW, Ghosh S, Walker IA, Carter A, Kerr L, Sharples L, Sebel PS, Bonke B, Winograd E (1993). Intraoperative positive therapeutic suggestions improve immediate postoperative recovery following cardiac surgery. Memory and awareness in anesthesia.

[48] Evans C, Richardson PH (1988). Improved recovery and reduced postoperative stay after therapeutic suggestions during general anaesthesia. Lancet.

[49] Boeke S, Bonke B, Bouwhuis-Hoogerwerf ML, Bovill JG, Zwaveling A (1988). Effects of sounds presented during general anaesthesia on postoperative course. Br J Anaesth.

[50] Bonke B, Schmitz PIM, Verhage F, Zwaveling A (1986). Clinical study of so- called unconscious perception during general anaesthesia. Br J Anaesth.

[51] Caseley-Rondi G, Merikle PM, Bowers KS (1994). Unconscious cognition in the context of general anesthesia. Conscious Cogn.

[52] Cowan GS, Buffington CK, Cowan GS, Hathaway D (2001). Assessment of the effects of a taped cognitive behavior message on postoperative complications (therapeutic suggestions under anesthesia). Obes Surg.

[53] De Houwer J, Demeyere R, Verhamme B, Eelen P, Bonke B, Bovill JG, Moerman N (1996). Intra- and post-operative effects of information presented during CABG surgery with sufentanil anaesthesia. Memory and awareness in anesthesia III.

[54] Eberhart LHJ, Döring HJ, Holzrichter P, Roscher R, Seeling W (1998). Therapeutic suggestions given during neurolept-anaesthesia decrease post-operative nausea and vomiting. Eur J Anaesthesiol.

[55] Furlong M, Bonke B, Fitch W, Millar K (1990). Positive suggestions presented during anaesthesia. Memory and awareness in anaesthesia.

[56] Furlong M, Read C, Sebel PS, Bonke B, Winograd E (1993). Therapeutic suggestions during general anesthesia. Memory and awareness in anesthesia.

[57] Lebovits AH, Twersky R, McEwan B (1999). Intraoperative therapeutic suggestions in day-case surgery: are there benefits for postoperative outcome. Br J Anaesth.

[58] Liu WHD, Standen PJ, Aitkenhead AR (1992). Therapeutic suggestions during general anaesthesia in patients undergoing hysterectomy. Br J Anaesth.

[59] Liu WDH, Standen PJ, Aitkenhead AR, Sebel PS, Bonke B, Winograd E (1993). The influence of intraoperative therapeutic suggestions on postoperative recovery after surgical repair of fractured neck of femur. Memory and awareness in anesthesia.

[60] Maroof M, Ahmed SM, Khan RM, Bano SJ, Haque AW (1997). Intra-operative suggestions reduce incidence of post hysterectomy emesis. J Pak Med Assoc.

[61] McLintock TTC, Aitken H, Downie CFA, Kenny GNC (1990). Postoperative analgesic requirements in patients exposed to positive intraoperative suggestions. Br Med J.

[62] McWilliams JL (1990). Using hypnotic suggestions to reduce postoperative nausea and pain following lumbar laminectomies [dissertation].

[63] Melzack R, Germain M, Belanger E, Fuchs PN, Swick R (1996). Positive intrasurgical suggestion fails to affect postsurgical pain. J Pain Symptom Manage.

[64] Moix J, Bayés R, Burrel L, Casas JM, Bonke B, Bovill JG, Moerman N (1996). Effects of intraoperative suggestions on intra- and postoperative variables: Preliminary report. Memory and awareness in anaesthesia III.

[65] Münch F, Zug H-D, Bonke B, Fitch W, Millar K (1990). Do intraoperative suggestions prevent nausea and vomiting in thyroidectomy-patients? An experimental study. Memory and awareness in anaesthesia.

[66] Nilsson U, Rawal N, Unestahl LE, Zetterberg C, Unosson M (2001). Improved recovery after music and therapeutic suggestions during general anaesthesia: a double-blind randomised controlled trial. Acta Anaesthesiol Scand.

[67] Oddby-Muhrbeck E, Jakobsson J, Enquist B (1995). Implicit processing and therapeutic suggestion during balanced anaesthesia. Acta Anaesthesiol Scand.

[68] Rosenberg JI (1992). Postoperative recovery and cost-benefit of an audiotape played to patients under general anesthesia [dissertation].

[69] Steinberg ME, Hord AH, Reed B, Sebel PS, Sebel PS, Bonke B, Winograd E (1993). Study of the effect of intra- operative suggestion on postoperative analgesia and well-being. Memory and awareness in anesthesia.

[70] van der Laan WH, van Leeuwen BL, Sebel PS, Winograd E, Baumann P, Bonke B (1996). Therapeutic suggestion has no effect on postoperative morphine requirements. Anesth Analg.

[71] Woo R, Seltzer JL, Marr A (1987). The lack of response to suggestion under controlled surgical anesthesia. Acta Anaesthesiol Scand.

