# Supplementary material for: Efficacy of therapeutic suggestions under general anesthesia: a systematic review and meta-analysis of randomized controlled trials
Source: BMC Anesthesiol. 2016 Dec 22;16:125. doi: 10.1186/s12871-016-0292-0 (PMC5178078; doi:10.1186/s12871-016-0292-0)
Supplement: Additional file 1: Table S1. — Measures of outcomes. (DOCX 15 kb) [file 12871_2016_292_MOESM1_ESM.docx]

Supplementary table 1. Measures of outcomes

| Outcome category | Outcome construct | Measurement |
| --- | --- | --- |
| Mental distress | Anxiety | State Trait Anxiety Inventory (STAI, state anxiety subscale); VAS |
|  | Well-being | NRS |
|  | Mood | Profile of Mood Scale (POMS); NRS; VAS |
|  | Relaxation | NRS |
| Pain intensity |  | VAS; NRS; number of patients reporting pain; McGill Pain Questionnaire (MPQ) |
| Medication | Use of antiemetics | Number of patients receiving medication; dosage of medication received |
|  | Use of analgesics | Number of patients receiving medication; dosage received; PCA |
| Recovery | Nausea | NRS; VAS; number of patients with nausea |
|  | Vomiting | NRS; VAS; number of patients vomiting; number of times patient vomited |
|  | Fluid replacement | Dosage |
|  | General recovery | NRS (rating by nurse); Patient Self-rating Questionnaire of Recovery |
|  | Mobility | NRS; VAS; number of patients with mobility problems; mobilization score by nurse; time from surgery until patient could sit, stand, walk without assistance; Hathaway Postoperative Regimen Checklist (subscale mobility) |
|  | Turning | Hathaway Postoperative Regimen Checklist (subscale turning) |
|  | Coughing | Hathaway Postoperative Regimen Checklist (subscale coughing) |
|  | Breathing | Hathaway Postoperative Regimen Checklist (subscale breathing) |
|  | Pyrexia | Number of half-days with elevated temperature |
|  | Bowel difficulties | Number of patients with bowel difficulties |
|  | Flatulence | VAS |
|  | Micturation | Number of patients with urinary problems |
|  | Complications | Number of patients with complications |
|  | Wound healing | Number of patients with wound infection |
|  | Side effects | Number of patients experiencing side effects |
|  | Activities of daily living | Activities of daily living scale (ADL) |
|  | Fatigue | NRS |
|  | Digestion | VAS |
|  | Appetite | VAS |
|  | Sleep | VAS |
| Length of procedure |  | Medical record |
| Physiological parameters | Blood pressure | Medical record |
|  | Blood loss | Medical record |
|  | Noradrenaline | Medical record |
|  | Adrenaline | Medical record |
|  | Heart rate | Medical record |

VAS = visual analogue scale; NRS = numeric rating scale
