# Supplementary material for: Efficacy of therapeutic suggestions under general anesthesia: a systematic review and meta-analysis of randomized controlled trials
Source: BMC Anesthesiol. 2016 Dec 22;16:125. doi: 10.1186/s12871-016-0292-0 (PMC5178078; doi:10.1186/s12871-016-0292-0)
Supplement: Additional file 2: Table S2. — Risk of bias in individual studies. (DOCX 17 kb) [file 12871_2016_292_MOESM2_ESM.docx]

Additional file 2: Table S2. Risk of bias in individual studies

| Study | Random sequence generation | Allocation concealment | Blinding of participants | Blinding of medical personnel | Blinding of outcome assessment | Incomplete outcome data | Selective reporting |
| --- | --- | --- | --- | --- | --- | --- | --- |
| Bethune 1993 | ? | ? | + | + | + | ? | ? |
| Block 1991 | ? | ? | + | + | + | + | ? |
| Boeke 1988 | ? | ? | + | + | + | + | ? |
| Bonke 1986 | ? | ? | + | + | + | + | ? |
| Caseley-Rondi 1994 | ? | ? | + | + | + | ? | ? |
| Cowan 2001 | ? | ? | + | + | + | + | ? |
| Dawson 2001 | + | ? | + | + | + | ? | ? |
| De Houwer 1996 | ? | ? | + | + | + | + | ? |
| Eberhart 1998 | ? | ? | + | + | + | + | ? |
| Evans & Richardson 1988 | ? | ? | + | + | + | ? | ? |
| Furlong & Read 1993 | ? | ? | + | ? | + | + | ? |
| Furlong 1990 | ? | ? | + | + | + | + | ? |
| Jelicic 1993 | ? | ? | + | + | + | + | ? |
| Korunka 1992 | ? | ? | + | + | + | + | ? |
| Lebovits 1999 | + | ? | + | + | + | + | ? |
| Liu 1992 | ? | ? | + | + | + | + | ? |
| Liu 1993 | ? | ? | ? | + | +/?* | ? | ? |
| Maroof 1997 | ? | ? | ? | ? | + | + | ? |
| Mastropietro 1998 | + | ? | ? | + | ? | + | ? |
| Mc Lintock 1990 | ? | ? | + | + | + | + | ? |
| McWilliams 1990 | + | + | + | + | + | ? | ? |
| Melzack 1996 | + | ? | + | + | + | + | ? |
| Moix 1996 | ? | ? | + | + | + | ? | ? |
| Münch & Zug 1990 | ? | ? | + | + | + | ? | ? |
| Nilsson 2001 | + | ? | + | + | + | ? | ? |
| Oddby-Muhrbeck 1995 | ? | ? | ? | ? | ? | + | ? |
| Pearson 1961 | ? | ? | + | + | + | ? | ? |
| Rosenberg 1992 | ? | ? | + | + | + | ? | ? |
| Steinberg 1993 | ? | ? | + | + | + | ? | ? |
| Van der Laan 1996 | ? | ? | + | + | + | + | ? |
| Williams 1994 | ? | ? | + | ? | + | + | ? |
| Woo 1987 | ? | ? | ? | ? | + | ? | ? |

+ low risk of bias, ? unclear risk of bias; * rating dependent on outcome variable
