# Supplementary figures and images for: Efficacy of therapeutic suggestions under general anesthesia: a systematic review and meta-analysis of randomized controlled trials
Source: BMC Anesthesiol. 2016 Dec 22;16:125. doi: 10.1186/s12871-016-0292-0 (PMC5178078; doi:10.1186/s12871-016-0292-0)

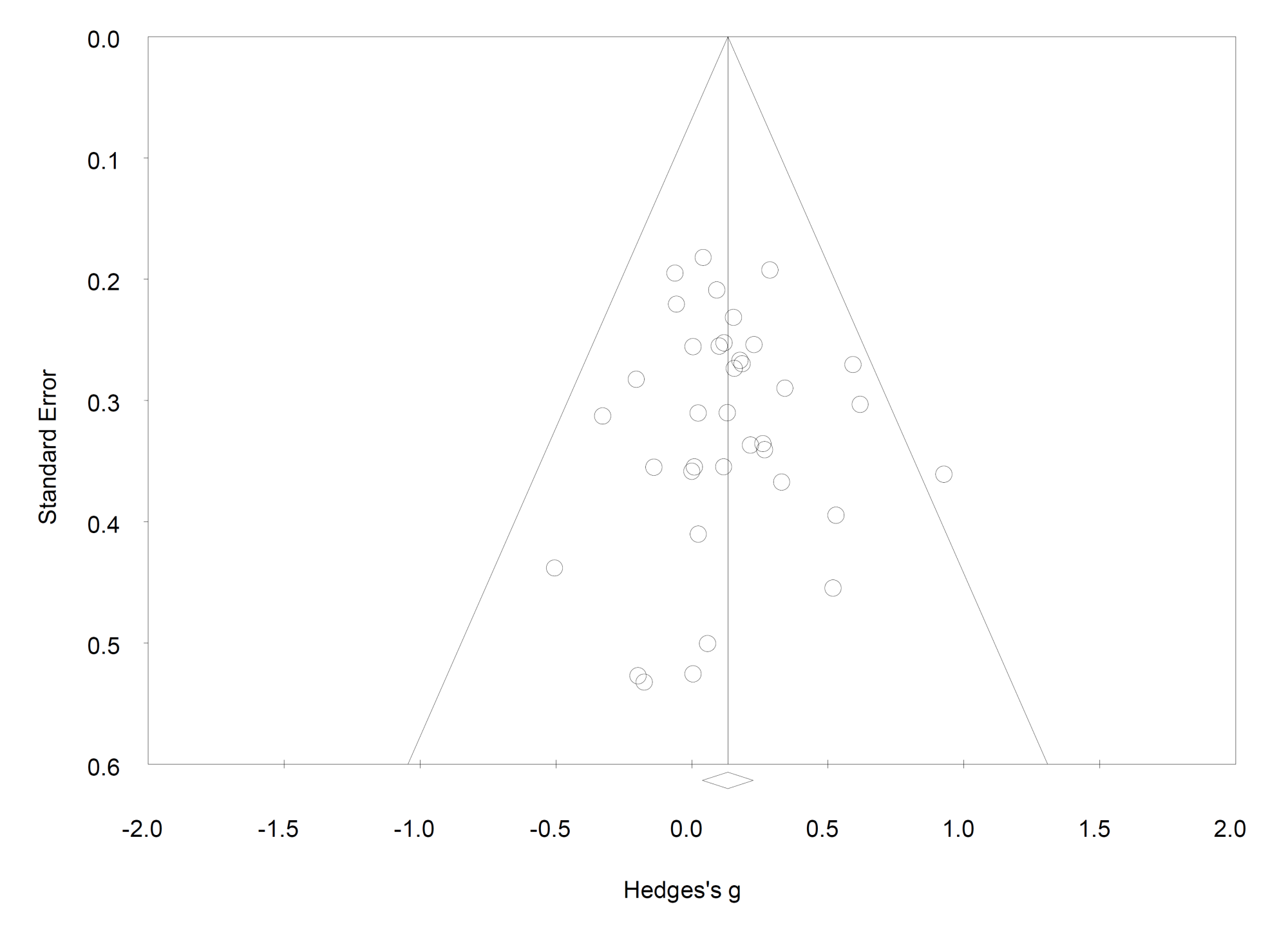

Supplement: Additional file 3: Figure S1. — Funnel plot of Hedges’ g against its standard error for all outcomes. (TIF 302 kb) [file 12871_2016_292_MOESM3_ESM.tif]
