# Supplementary material for: Efficacy of therapeutic suggestions under general anesthesia: a systematic review and meta-analysis of randomized controlled trials
Source: BMC Anesthesiol. 2016 Dec 22;16:125. doi: 10.1186/s12871-016-0292-0 (PMC5178078; doi:10.1186/s12871-016-0292-0)
Supplement: Additional file 4: Table S3. — Results of sensitivity analyses for the exclusion of approximated effect sizes and effect sizes which were set to zero, and for the exclusion of small samples. (DOCX 18 kb) [file 12871_2016_292_MOESM4_ESM.docx]

Additional file 4: Table S3. Results of sensitivity analyses for the exclusion of approximated effect sizes and effect sizes which were set to zero, and for the exclusion of small samples

|  | Hedges´ g | 95% CI | k | p | Heterogeneity | | |
| --- | --- | --- | --- | --- | --- | --- | --- |
|  |  |  |  |  | Q (df) | p | I^2^ |
| **Mental distress** |  |  |  |  |  |  |  |
| all effect sizes | 0.03 | -0.11; 0.16 | 18 | .721 | 10.08 (17) | .900 | 0.0 |
| approximated excluded | 0.001 | -0.18; 0.18 | 11 | .991 | 6.23 (10) | .795 | 0.0 |
| sample size N ≥ 20 per group | 0.03 | -0.13; 0.19 | 12 | .686 | 3.93 (11) | .972 | 0.0 |
| **Pain intensity** |  |  |  |  |  |  |  |
| all effect sizes | 0.04 | -0.04; 0.12 | 24 | .317 | 16.90 (23) | .817 | 0.0 |
| approximated excluded | 0.06 | -0.06; 0.18 | 14 | .315 | 14.51 (13) | .339 | 10.4 |
| sample size N ≥ 20 per group | 0.05 | -0.03; 0.13 | 22 | .247 | 15.08 (21) | .819 | 0.0 |
| **Medication** |  |  |  |  |  |  |  |
| all effect sizes | 0.19 | 0.09; 0.29 | 27 | <.001 | 24.55 (26) | .544 | 0.0 |
| approximated excluded | 0.20 | 0.09; 0.31 | 21 | .001 | 22.19 (20) | .330 | 9.9 |
| sample size N ≥ 20 per group | 0.19 | 0.09; 0.29 | 24 | <.001 | 21.06 (23) | .577 | 0.0 |
| Antiemetic use |  |  |  |  |  |  |  |
| all effect sizes | 0.22 | -0.003; 0.45 | 9 | .053 | 13.01 (8) | .111 | 38.5 |
| approximated excluded | 0.44 | -0.08; 0.96 | 4 | .100 | 11.30 (3) | .010 | 73.5 |
| sample size N ≥ 20 per group | no study excluded | | | | | | |
| Analgesic use |  |  |  |  |  |  |  |
| all effect sizes | 0.16 | 0.06; 0.26 | 26 | .002 | 21.37 (25) | .672 | 0.0 |
| approximated excluded | 0.20 | 0.08; 0.32 | 17 | .001 | 14.88 (16) | .533 | 0.0 |
| sample size N ≥ 20 per group | 0.15 | 0.05; 0.25 | 23 | .003 | 17.70 (22) | .724 | 0.0 |
| **Recovery** |  |  |  |  |  |  |  |
| all effect sizes | 0.14 | 0.03; 0.25 | 27 | .016 | 23.65 (26) | .596 | 0.0 |
| approximated excluded | 0.15 | 0.03; 0.26 | 25 | .011 | 20.29 (24) | .680 | 0.0 |
| sample size N ≥ 20 per group | 0.13 | 0.01; 0.25 | 21 | .030 | 20.29 (20) | .440 | 1.4 |
| PONV |  |  |  |  |  |  |  |
| all effect sizes | 0.21 | 0.07; 0.36 | 21 | .004 | 23.75 (20) | .254 | 15.8 |
| approximated excluded | 0.27 | 0.10; 0.43 | 18 | .001 | 20.99 (17) | .227 | 19.0 |
| sample size N ≥ 20 per group | 0.22 | 0.05; 0.39 | 17 | .012 | 23.12 (16) | .111 | 30.8 |
| Recovery (all other outcomes) |  |  |  |  |  |  |  |
| all effect sizes | 0.11 | -0.01; 0.24 | 19 | .073 | 13.71 (18) | .748 | 0.0 |
| approximated excluded | 0.21 | 0.06; 0.35 | 14 | .005 | 12.56 (13) | .483 | 0.0 |
| sample size N ≥ 20 per group | 0.10 | -0.03;0.24 | 14 | .130 | 9.74 (13) | .715 | 0.0 |

PONV = postoperative nausea and vomiting
