# Supplementary material for: Efficacy of therapeutic suggestions under general anesthesia: a systematic review and meta-analysis of randomized controlled trials
Source: BMC Anesthesiol. 2016 Dec 22;16:125. doi: 10.1186/s12871-016-0292-0 (PMC5178078; doi:10.1186/s12871-016-0292-0)
Supplement: Additional file 5: Table S4. — Results of subgroup analyses for PONV and antiemetic use. (DOCX 20 kb) [file 12871_2016_292_MOESM5_ESM.docx]

Additional file 5: Table S4. Results of subgroup analyses for PONV and antiemetic use

|  | PONV | | | | | | | | Antiemetic use | | | | | | | |
| --- | --- | --- | --- | --- | --- | --- | --- | --- | --- | --- | --- | --- | --- | --- | --- | --- |
|  | g | 95% CI | k | p | Heterogeneity | | | p^#^ | g | 95% CI | k | p | Heterogeneity | | | p^#^ |
|  |  |  |  |  | Q (df) | p | I^2^ |  |  |  |  |  | Q (df) | p | I^2^ |  |
| Specificity of suggestions |  |  |  |  |  |  |  | .148 |  |  |  |  |  |  |  | .664 |
| “no sickness” suggestions | 0.29 | 0.09; 0.49 | 14 | .004 | 18.24 (13) | .149 | 28.7 |  | 0.26 | -0.03; 0.55 | 7 | .078 | 12.90 (6) | .045 | 53.5 |  |
| suggestions not related to PONV | 0.08 | -0.14; 0.29 | 7 | .476 | 3.16 (6) | .788 | 0.0 |  | 0.15 | -0.24; 0.55 | 2 | .450 | 0.09 (1) | .765 | 0.0 |  |
| Affirmativity |  |  |  |  |  |  |  | .700 |  |  |  |  |  |  |  | .112 |
| affirmative suggestions only | 0.31 | 0.03; 0.59 | 4 | .031 | 0.25 (3) | .970 | 0.0 |  | 0.03 | -0.27; 0.33 | 2 | .849 | 0.69 (1) | .407 | 0.0 |  |
| affirmative and non-affirmative | 0.23 | -0.05; 0.52 | 11 | .113 | 20.49 (10) | .025 | 51.2 |  | 0.39 | 0.06; 0.71 | 6 | .019 | 8.05 (5) | .154 | 37.9 |  |
| Anaesthesia |  |  |  |  |  |  |  | .505 |  |  |  |  |  |  |  | .353 |
| Neuroleptanesthesia | 0.29 | 0.04;0.55 | 7 | .025 | 6.31 (6) | .390 | 4.9 |  | 0.35 | -0.04; 0.74 | 4 | .076 | 4.75 (3) | .191 | 36.8 |  |
| Inhalation or intravenous anaesthesia | 0.19 | 0.01; 0.36 | 14 | .040 | 16.78 (13) | .210 | 22.5 |  | 0.13 | -0.13; 0.39 | 5 | .321 | 6.10 (4) | .192 | 34.4 |  |
| Random sequence generation |  |  |  |  |  |  |  | .321 |  |  |  |  |  |  |  | .545 |
| low risk of bias | 0.06 | -0.27; 0.39 | 5 | .728 | 1.45 (4) | .836 | 0.0 |  | 0.16 | -0.14; 0.46 | 4 | .289 | 0.11 (3) | .990 | 0.0 |  |
| unclear risk of bias | 0.25 | 0.08; 0.42 | 16 | .005 | 21.37 (15) | .126 | 29.8 |  | 0.31 | -0.08; 0.71 | 5 | .115 | 12.88 (4) | .012 | 68.9 |  |
| Incomplete outcome data |  |  |  |  |  |  |  | .061 |  |  |  |  |  |  |  | .251 |
| low risk of bias | 0.35 | 0.11; 0.59 | 11 | .004 | 17.60 (10) | .062 | 43.2 |  | 0.58 | -0.20; 1.35 | 3 | .145 | 10.99 (2) | .004 | 81.8 |  |
| unclear risk of bias | 0.06 | -0.14; 0.25 | 10 | .557 | 1.74 (9) | .995 | 0.0 |  | 0.11 | -0.10; 0.31 | 6 | .314 | 0.64 (5) | .986 | 0.0 |  |

PONV = postoperative nausea and vomiting; ^#^ Test of effect size differences between subgroups (p-value).
